# Economic utility of Colombian Romosinuano cattle

**DOI:** 10.1007/s11250-024-04066-z

**Published:** 2024-07-20

**Authors:** Juana Moncaleano-Vega, Alejandro Amaya, Carlos Martínez, William Burgos-Paz, Mario Cerón-Muñoz

**Affiliations:** 1 Facultad de Ciencias Agrarias, Doctorado en Ciencias Animales, Universidad de Antioquia, Medellín, Colombia; 2 Universidad de Ciencias Aplicadas y Ambientales U.D.C.A, Programa de Zootecnia, Bogotá, Colombia; 3 Departamento de Producción Animal, Facultad de Medicina Veterinaria y Zootecnia, Universidad Nacional de Colombia, Bogotá, Colombia; 4 Corporación Colombiana de Investigación Agropecuaria-AGROSAVIA, Centro de Investigación Turipaná, Km 13 Vía Montería-Cereté, Córdoba, Colombia; 5 Grupo de Investigación GAMMA, Facultad de Ciencias Agrarias, Universidad de Antioquia, Medellín, Colombia; 6 Facultad de Medicina Veterinaria y Zootecnia, Universidad del Tolima, Ibagué, Colombia

**Keywords:** Bioeconomics, Creole cattle, Genetic improvement

## Abstract

The objective of this study was to quantify the economic utility in Romosinuano production systems by developing a bioeconomic model assumed cow-calf, cow-calf plus stocker (CCPS), and complete cycle operations. Each system produced males for sale and females for replacement. Input parameters were established from breed data collected by AGROSAVIA. Revenues were estimated using the official cattle price, and production costs were quantified per activity. In the results, for cow-calf operations, the maximum economic utility was 244.12 USD. CCPS, yielded 231.86 USD, and Complete cycle, 268.94 USD. The genetic progress per generation for W240, W480, W24 and CI was + 3.8 kg, + 5 kg, + 5.9 kg, and -1 d, respectively. The price of livestock was the sensitized variable with the greatest impact on maximum economic utility (± 118.64 USD to ± 155.44 USD), followed by mineral supplementation (16.31 USD to ± 37.34 USD). The sensitized variables with the lowest impact were food (± 1.62 USD to ± 1.8 USD) and health plan supplies (± 6.03 USD to ± 9.13 USD). It is concluded that economic utility defined as a composite trait influenced by the characteristics that shape it favors genetic progress and the identification of animals with optimal performance in different bovine production systems.

## Introduction

In Colombia, the genetic improvement programs of the Romosinuano breed are based on performance tests and use genetic and genomic information to estimate the genetic merit of breeding animals for economically important traits (Cañas-Álvarez et al. 2023). This implies assessing the economic utility of animal performance (Spangler and Weaber 2017; Rowan 2022). However, quantifying economic utility while considering the productive efficiency of animals in extensive grazing systems poses certain challenges related to the classification of production costs and income per cow, aspects crucial for evaluating the profitability, sustainability, and success of the livestock enterprise (Spangler 2015).

In cattle production systems, the activity-based costing (ABC) method can describe and allocate costs to activities using indicators (kilograms, liters, days, etc.) generated directly in activities that consume resources and induce costs (Valera and Morrillo 2009; Eslava and Parra 2019). Thus, the cost of the finished product (calf weight) is determined by the costs necessary for its production (Buitrago 2020). However, revenues fluctuate based on cattle prices and the supply and demand for calves. For example, the demand for calves may influence decisions regarding whether to retain females for reproduction (capital asset) or send them for slaughter (PEGA 2006).

Bioeconomic models are a strategy to integrate costs, income, physical resources, management and to derive economic values under productive and economic analysis (Pravia et al. 2014; López-Paredes et al. 2017; Amaya et al. 2022). These models can be adapted to estimate the economic contribution per animal. This adaptation involves creating grids than combine phenotypic measures of economically relevant traits (ERT). By incorporating these grids as part of the input parameters, the most profitable combinations can be recorded and used to identify the most efficient animals. With this premise, our objective was to quantify the economic utility by adapting bioeconomic models to three types of Romosinuano breed production systems.

## Materials and methods

Adjusted weaning weights at 240 days (W240), at 480 days (W480), and 24 months (W24) of age, calving interval (CI), and age at first calving (AFC) were used as ERT. Data records were obtained from Banco de Germoplasma del Ganado Romosinuano compiled by the Colombian Agricultural Research Corporation – AGROSAVIA. We considered three Romosinuano production systems: cow-calf, cow-calf plus stocker (CCPS), and complete cycle operations. The end goal was produced males for sale of eight-month age, 16-month age, and 24-month age, respectively; the female will stay on the farm as part of the breeding system and sell the surplus. Each system had 100 cows from 1 to 10 calvings, in rotational extensive grazing *Brachiaria spp*, occupied for 5 days and 63 days of rest, producing 4 tons of forage per hectare, minus 30% loss due to trampling to define the available grazing forage (AGF), and daily supply of mineralized salt. Other parameters, such as replacement rate, animal culling, and survival rates, number of calving per year, and the proportion of female and male births, are presented in Table 1.

**Table 1:** Herd structure and input parameters of the bioeconomic model of the Romosinuano breed

Item	Unit
First calving cow	22
Second calving cow	18
Cow from third to tenth calving	60
Total number of cows	100
Lactation length days	240
240-day milk production in liters	960
Male | Females Birth Ratio	0.50
Calving per cow-year (365/CI)	0.93
Pre-weaning survival rate	94%
Post-Weaning survival rate	98%
Percentage of survival 24-month age	98%
Adult Survival Rate	99%
Annual Replacements by Category	13
Annual Cows discarded	13

The dry matter (DM) required to meet energy requirements for maintenance, growth, lactation, and the last third of gestation in each system (${dmt}_{i}$, where *dmt* is dry matter total, and *i* is cow-calf, CCPS, or complete cycle) was calculated using the National Research Council (NRC, 2021) equations, assuming a net energy for lactation of 0.95 Mcal/kg DM and net energy for growth of 0.57 Mcal/kg DM. The average intake per cow was calculated $I:{dmt}_{i}/100$, and the number of hectares ($ha$) per production system: ${ha=dmt}_{i}/AGF$.

To identify drivers and assign unit costs, Romosinuano cattle breeders were surveyed using the following key questions (Spangler and Weaber 2017): What are breeding or marketing objectives? Which trait directly impact the profitability of farming? What environmental or management limitations exist on the farm that influence animal performance in a specific trait? Are replacement females purchased or bred? Subsequently, three cost-centers were defined: feeding, supplementation, and health plan.

The feeding cost-center was defined as: ${a}_{i}={dmt}_{i}*ca$, where *ca* was the unit cost of kg DM in the grazing, derived from pasture management investment. The supplementation cost-center was defined as: ${u}_{i}={sv}_{i}*csv+{sm}_{i}*csm+c{Fa}_{i}*{Fa}_{i}$. Here, ${sv}_{i}$ was the amount of salt supplied to females, ${sm}_{i}$ the amount of salt supplied to males, and ${Fa}_{i}$ kg of silage supplied in the *i*-th production system. The cost of one kilogram of salt for females (csv) and for males (csm) depends on the type of salt supplied. Additionally, the cost of one kilogram of silage DM (${cFa}_{i}$) depends on the investment allocated to on-farm production. The health plan cost-center was defined as: ${p}_{ik}={\Sigma }_{k=1}^{5}{k}_{ik}*{USD}_{k}$, where *k* represented: cow (1), calf (2), rearing (3), female (4), and replacement (5) in the *i*-th production system, and the value in USD at cost per animal in each category.

The cost per cow (C) excluding grazing feeding costs, were calculated as follows: $C=({u}_{i}/100+{p}_{i}/100)$. The revenues per cow (B) were defined as the total live weight produced multiplied by the official cattle price, divided by the number of cows in production: $B={\Sigma }_{j=1}^{7}{(kg}_{ij}*{USD}_{j})$​​, where the *j* represents weaned calf (1), weaned heifer (2), 16-month-old male (3), 16-month-old female (4), 24-month-old male (5), 24-month-old female (6), cull cow (7), and the value in USD depended on the price per kg for the respective category (Table 2).

**Table 2:** Price of live cattle to calculate income in Romosinuano production systems

Category	Males^a^	Females^a^
8 months	1.95	1.95
16 months	1.85	1.83
24 months	1.66	1.64
Discard cow		1.73

^a^USD per kg: information based on prices from the Official Gazette no. 35 of Central Ganadera, Medellín, 2023

The base economic utility (${\pi }_{i}$) of the *i*-th production system was calculated using each trait average (Garrick 2002). The maximum economic utility per system (${\pi }_{{i}_{m}})$ and the phenotypic measures modeling it were determined by assigning a phenotypic value to every ${x.}_{i}$​ (where ${x}_{\bullet i}$ is the trait involved in the *i*-th production system), located at percentiles (*q*) 48, 50, and 54 (${x}_{{\bullet q}_{48}}\le {x}_{{\bullet q}_{50}}\le {x}_{{\bullet q}_{54}}$​​) referred to in Table 3.

**Table 3:** Phenotypes of economically relevant characteristics of the production systems of the Romosinuano breed

Item	Registers	Histogram	Rank	Average	Median $\left({q}_{50}\right)$	Coefficient of variation (%)	Percentiles (q)
W240 (kg)	5186	Figure 1	[70 – 270]	162.6 ± 35.56	162	21.87	${q}_{54}=$ 166
W480 (kg)	3828	Figure 2	[93 – 370]	197.32 ± 38.62	196	19.57	${q}_{54}=$ 201
W24 (kg)	1682	Figure 3	[152 – 439]	295.93 ± 52.02	294	17.57	${q}_{54}=$ 300
AFC (d)	1595	Figure 4	[736 – 1775]	1187.26 ± 184.67	1132	15.55	${q}_{48}=$ 1127
CI (d)	2804	Figure 5	[330 – 495]	388.33 ± 28.71	383	7.39	${q}_{48}=$ 382

Descriptive measures for: W240 (weaning weight adjusted to 240 days); W480 (weight adjusted to 480 days of age); W24 (weight 24 months); AFC (age at first calving); CI (calving interval). Unit of measurement: kilograms (kg), and day (d). ${q}_{54}$: 54th percentile, ${q}_{50}$: 50th percentile, ${q}_{48}$: 48th percentile. Figures 1, 2, 3, 4 and 5 show right-skewed distributions for each ERT, with respective skewness values of 0.089, 0.067, 0.044, 0.72, and 0.54. CI was the characteristic with the lowest coefficient of variation (7.39%), and W240 was the weight with the highest coefficient of variation (21.87%) in the Romosinuano breed.

These ranges were utilized to generate grids of values represented by vectors (${y}_{i}$), which were then combined and simultaneously executed in the programmed model. In this process, the equation suggested by Garrick (2002) for extensive cow-calf grazing systems was adapted:

$${\pi }_{{i}_{m}}=\left({B}_{{y}_{i}}-{C}_{{y}_{i}}\right)*\left(\frac{{Fdp}_{i}+{Fa}_{i}}{{I}_{i}}\right)-{Fa}_{yi}*c{Fa}_{i}$$

Assuming that ${\pi }_{{i}_{m}}$ in the next generation is achieved, and its phenotypic measures are the expected changes, the genetic progress (${\Delta x}_{\bullet i}$​) was estimated using the phenotypic measures for the maximum economic utility (${x}_{{\bullet i}_{m}}$) minus the vector of means: ${\Delta x}_{\bullet i}=({x}_{{\bullet i}_{m}}-{x_\bullet{{_q}_{50}}})$​​). To find the average phenotypic measures of the parents to be selected (${x}_{{\bullet i}_{s}}$), the ${\Delta x}_{\bullet i}$; ${x_\bullet}_{{q}_{50}}$; and ${{h}^{2}}_{x_\bullet}$ were used: ${x}_{{\bullet i}_{s}}={\Delta x}_{\bullet i}/{{h}^{2}}_{x_\bullet}+{x}_{{\bullet q}_{50}}$ (Lush 1937). All the described procedures were integrated into a R-project script (R Core Team 2023). Finally, to measure genetic progress per year, the generational interval was averaged across four genetic pathways: sire-son, sire-daughter, dam-son, and dam-daughter using the PEDIG software (Boichard 2002), and heritability was estimated using the AIREMLf90 program of BLUPf90 (Misztal et al. 2018).

To quantify and identify the magnitude of changes in maximum economic utility if market conditions change in the base production system, a sensitivity analysis was performed. To this end, modifications of 40% (increase and decrease) were applied, separately, to the price per kg of live weight of male calves, surplus heifers and cull cows, mineral supplements cost, health plan cost and to the DM prices of the feed, preserving the other input parameters, and phenotypic measures of the ERT that generated the maximum economic utility. These modifications were integrated into the programming code of each model, executed again and the sensitized economic utilities of each system were obtained.

## Results

The base utility and maximum economic utility for each system are shown in Table 4. The maximum utility was determined empirically by exploring the grids constructed from ERT previously defined. The AFC was always equal to the median in all three production systems (1132 d).

**Table 4:** Phenotypes of the Romosinuano breed related to the maximum economic utility per system

Systems	Utility (USD)		Phenotypes			
	Base	Maxim	W240 (kg)	W480 (kg)	W24 (kg)	CI (d)
Cow-calf	241.99	**244.12**	**165.8**			**382**
Cow-calf plus stocker	227.03	**231.86**	**162.6**	**201**		**382**
Complete cycle	264.69	**268.94**	**162.4**	**201**	**299.9**	**383**

W240: weaning weight adjusted to 240 days. W480: weight adjusted to 480 days of age. W24: weight 24 months old. CI: calving interval. In bold, phenotypes related to maximum economic utility per system.

For cow-calf system, the maximum utility (244.12 USD) was associated with W240, AFC, and CI traits at ${q}_{54}$, ${q}_{50}$, and ${q}_{48}$, respectively (Tables 3 and Table 4). For CCPS, the maximum utility, 231.86 USD, was achieved with 162.6 kg at W240; 201 kg at W480; IC of 382 d and AFC of 1132 d (Table 4). For complete cycle, the optimal utility, 268.94 USD, was observed when W240 = 162.4 kg; W480 = 201 kg, and W24 = 299.9 kg; AFC of 1132 d and CI of 383 d. In this system, the genetic progress was achieved with W24 of + 0.713 kg per year, and the average phenotype of parents should be 295.55 kg for W24; AFC and CI should be maintained at ${q}_{50}$, respectively (Table 4).

The genetic progress per generation and per year for each CER, its heritability, and the average phenotype of the parents of the next generation are presented in Table 5. The average generational interval was 8.27 years.

**Table 5:** Heritability and Genetic Progress for Productive Traits in Romosinuano Breed

Parameter	W240 (kg)	W480 (kg)	W24 (kg)	CI (d)
Genetic Progress by Generation	3.8	5	5.99	-1
Genetic progress by year	0.459	0.604	0.713	-0.12
Heritability	0.21^a^	0.42	0.46	0.031
Average Parent Phenotype	180.1	207.9	304.87	351

*W240* weaning weight adjusted to 240 days, *W480* weight adjusted to 480 days of age, *W24* weight 24 months old, *CI* calving interval, *a* Total heritability

For cow-calf system, according to the combination of phenotypic measures of the ERT, maximum utility requires genetic progress per year for CI and W240 of -0.12 d (-1 d per generation) and + 0.459 kg (+ 3.8 kg per generation), respectively. With these results, the average phenotype of parents to select to maximize utility in the first year will be W240:164.18 kg; CI: 379 d; AFC equal to the median. For CCPS, the annual genetic progress that was established from the combination of the ERT that caused the maximum utility was: W480 of + 0.605 kg and -0.12 d in the CI. During the first year, the parents to be selected must have records for W480 of 197.43 kg, CI of 379 d, with AFC equal to the median. For complete cycle only required to improve the W24 with + 0.713 kg per year, and the average phenotype of the parents to be selected during the first year must register: 295.55 kg of W24, the AFC and the CI must be sustained at ${q}_{50}$, respectively (Table 5).

The results of the sensitivity analysis are presented in Table 6, indicating the effects of price variation (feed, mineralized salt, health plan and livestock price) on the maximum economic profit in the three livestock production systems of Romosinuano.

**Table 6:** Maximum economic utility and sensitized variables in Romosinuano production systems

	Sensitized variable							
System	Feeding		Mineralized salt		Health plan		Price per kg of live weight	
	-40	+ 40	-40	+ 40	-40	+ 40	-40	+ 40
Cow-calf	245.84	242.50	260.93	227.31	250.15	238.09	125.48	368.47
Cow-calf plus stocker	232.93	230.79	261.78	227.31	239.16	224.57	100.83	362.89
Complete cycle	270.35	267.54	306.28	231.60	278.07	259.82	113.5	424.39

The price per kg of live weight was the sensitized variable with the greatest impact on the maximum economic utility, with variations between ± 118.64 USD in the cow-calf system, ± 131.07 USD in CCPS, up to ± 155.44 USD in complete cycle; followed by changes in the price of mineral supplementation, with impacts of ± 16.31 USD, ± 29.92 USD and ± 37.34 USD, respectively. The sensitized variables with the lowest impact were feeding with ± 1.62 USD, ± 1.07 USD and ± 1.8 USD; and health plan with ± 6.03 USD, ± 7.3 USD, to ± 9.13 USD, in cow-calf system, CCPS and complete cycle, respectively.

Within the sensitized variables, the price change and the relationship with the maximum economic utility presented a linear trend. In this regard, the negative impact on the maximum economic utility was given by the prices of salt for replacements heifer from 8 to 16 months in cow-calf system and CCPS (-0.548 and -0.382, respectively), and for cull cows in complete cycle ( -0.457). Followed by the costs of the health plan for calving from 0 to 8 months in cow-calf system and CCPS (-0.452 and -0.312, respectively) and for replacements heifer from 8 to 16 months in complete cycle (-0.292).

## Discussion

The cost-centers described are characteristic of Colombian production systems based on grazing economy (González et al. 2022). Their definition, the animal resource, and the set of traits directly impacting income and costs made it possible to quantify the maximum economic utility in each production system (Mosnier et al. 2009; Mestra-Vargas et al. 2020).

The reduction in CI favored profitability in cow-calf system (0.532 USD) and complete cycle (0.654 USD) by improving income per cow, followed by the increase in kg of growth characteristics (0.395 USD, 0.104 USD, respectively), which is consistent with previous research by Roberts et al. (2015), López-Paredes et al. (2017) and Amaya et al. (2022). However, in this work no economic values were analyzed, the combination of the phenotypic measures of W240, W480, W24, CI and AFC was identified, for the first time, in the Romosinuano breed, which optimize the economic utility per cow, ha., year, between USD 2.13 and USD 4.82, in different production systems, adapting bioeconomic models (Garrick 2002; Wahinya et al. 2022) and using percentiles to create the combinations that assisted decision making (Zuliani et al. 2018). This result was used to delimit the genetic progress per year according to the resources of each production system (Abreu et al. 2018; Krupová et al. 2020; Wahinya et al. 2022).

The economic utility quantified per animal, based on the set of phenotypic measures that comprise it, could constitute a tool to reference those with optimal performance in different production environments (Notter 2013). This approach would allow the most outstanding individuals to be selected without the need to weight the ERT according to their importance or economic value, an aspect that represents one of the greatest challenges in the construction of selection indices (Spangler et al. 2017), although the AFC is high for beef production systems (Twomey and Cromie 2023), improving it would not only provide heifers, it would also change the age structure of the herd, as noted by Roberts et al. (2015). This change would be convenient to reduce the generation interval and cause favorable modifications of genetic progress in the population (Amaya et al. 2020).

In the sensitivity analysis, the scenarios provide evidence of the tolerance of Romosinuano's production systems to the rise in supplies livestock and the fall in prices per kg of live weight to produce animals of 8, 16 and 24 months of age. This behavior may be due to the fact that producers only depend on their grazing system for the production and sale of animals, with low investment in pasture management, offering additional food in times of forage scarcity, without being a generalized practice (Parra-Cortés and Magaña-Magaña 2021), grazing and grassland management are confirmed as the most profitable means to satisfy the energy requirements of Romosinuano livestock production systems (González et al. 2022).

In these scenarios, the negative impact of mineral supplementation could be associated with the number of animals in the production stage, which corresponds to replacement females in the category of 8 to 16 months that are not marketable, in addition to the consumption of cull cow, whose sales price per kg is the lowest on the market (Amaya et al. 2020). The changes associated with higher prices in the health plan may occur due to the use of adjuvants for healthy growth and development during the stages of greatest bovine growth. These costs that affect utility through category costs to non-marketable animals in these stages of production (Ramsey et al. 2005).

With respect to the changes in the price per kg of live weight and its impact on the maximum economic utility, the impact of the prices of the kg of cull cow on the utility of the three production systems (cow-calf system: 0.038; CCPS: 0.036 and complete cycle: 0.045) was greater than the impact of raising awareness about prices per kg of live weight of males or surplus females. Although the objective is not to fatten cull cows, it is evident that improving the sale price is more relevant due to the adult weight of the cows and the kg available per animal for sale (Kamilaris et al. 2020).

However, for future analysis, multidimensional sensitivity models could be considered, which allow exploring the relationship that exists between the characteristics, for example, between: CI, number of births per year, survival, price per kg of lives weight and supplies cost, to determine to what extent, in each production system, a fall in the price of livestock, a rise in cost supplies, as well as a decrease in the number of kg available for sale can be allowed (Ramsey et al. 2005; Kamilaris et al. 2020).

Bioeconomic models are integral part of precision agriculture, as they gather data from different sources, support decision making related to managing variations within a herd, and in maximizing yields, particularly when there are land limitations (Marote 2010). They allow adjustments to herd size and structure, energy requirements, pasture dry matter production, and ERT number (Mosnier et al. 2009; McClearn et al. 2020). Therefore, define acceptable percentiles ranges, which allow finding the phenotypes of the parents of the next generation, knowing the heritability of the characteristic, the input parameters, number and set of ERT, must be carried out in accordance with the defined production system, and these results should only serve as a basis for stablishing standards in the quantification of economic utility, (Zuliani et al. 2018), and how to use it in the design of sustainable genetic improvement objectives within a specific production system (Wahinya et al. 2022).

In conclusion, the quantification of economic utility using bioeconomic models that can be adapted to different production systems, and allows the projection of genetic progress per year from the phenotypic combinations that provide maximum economic utility, adjusting land use to the animal performance, could be considered to preselect Romosinuano cows with the objective of maximizing the expected genetic gain of net merit, if we define economic utility as a composite trait influenced by the characteristics that shape it. These results could be relevant to generate an indicator of productivity of Romosinuano cows, using their records for age at first calving, calving interval and weights at 8 months, 16 months, and 24 months of age, considering the relationship that exists among the traits. Finally, the strategic exploration of characteristics of economic interest in the production systems of the Romosinuano breed by implementing bioeconomic models is a valuable tool to demonstrate the productivity of these livestock systems.

## Data Availability

The datasets used and/or analyzed during this study are not publicly available due to AGROSAVIA institutional policies of and due to the confidentiality Romosinuano cattle breeders´ data.

## Code availability

The described procedures into in R-project script are available from the corresponding author on reasonable request.

## References

[CR1] Abreu SBC, Eler JP, Santana ML Jr, Mattos EC, Menezes IR, Ferraz JBS (2018) Genetic association between mature weight and early growth and heifer pregnancy traits in Nellore cattle. Livest Sci 211:61–65

[CR2] Amaya A, López PLC, Ramírez J (2022) Selection indexes to optimise genetic and economic progress in Colombian Blanco Orejinegro cattle. Livest Sci 263:105015. 10.1016/j.livsci.2022.105015

[CR3] Amaya A, Martínez R, Cerón-Muñoz M (2020) Parámetros genéticos para crecimiento y reproducción en ganado Simmental mediante parentesco por pedigrí y genómico. Revista MVZ Córdoba 25(1):1520. 10.21897/rmvz.1520

[CR4] Boichard D (2002) Pedig: A fortran package for pedigree analysis suited for large populations. In Proceedings of the 7th WorldCongress on Genetics Applied to Livestock Production. Montpellier, France, 19–23 August. https://www.researchgate.net/publication/375739173_Inbreeding_Depression_and_Purging_for_Meat_Performance_Traits_in_German_Sheep_Breeds. Accessed 05 Dec 2023

[CR5] Buitrago NDA (2020) Costos ABC para la producción de leche y carne en el municipio de Capitanejo, Santander. AGLALA 11(2):117–132 (ISSN 2215–7360)

[CR6] Cañas-Álvarez JJ, Ossa-Saraz GA, Garcés-Blanquiceth JL, Burgos-Paz WO (2023) Genealogical structure of the Colombian Romosinuano Creole cattle. Trop Anim Health Prod 55(5):292 37589774 10.1007/s11250-023-03694-1 PMC10435628

[CR7] Central Ganadera (2023) https://centralganadera.com/wp-content/uploads/2023/09/35-precios-oficiales-28-agosto-%2001-%20septiembre-2023_compressed.pdf

[CR8] Eslava ZRA, Parra GB (2019) Costos basados en actividades (ABC): análisis de los factores claves identificados en las investigaciones desarrolladas., 6to Simposio Internacional de Investigación en Ciencias Económicas, Administrativas y Contables – Sociedad y Desarrollo 2do Encuentro Internacional de Estudiantes de Ciencias Económicas, Administrativas y Contables Bogotá, 12, 13 y 14 de septiembre de 2019*.* unilibre.edu.co

[CR9] Garrick DJ (2002) Accounting for feed costs in improvement programmes for grazed dairy cattle. 7th World Congress on Genetics Applied to Livestock Production, August 19-23. Montpellier, France*.* https://www.researchgate.net/

[CR10] González A, Valderrama P, Zapata J, Fonseca M, Rodríguez J, Gómez J, Méndez D, Castro L, Toro Á (2022) Plan de Ordenamiento Productivo para la Cadena Cárnica Bovina en Colombia. Bogotá: UPRA. Recuperado de. https://upra.gov.co/es-co/Paginas/pop-carnica.aspx

[CR11] Kamilaris C, Dewhurst RJ, Vosough AB, Crosson P, Alexander P (2020) A bio-economic model for cost analysis of alternative management strategies in beef finishing systems. Agric Syst 180:102713. 10.1016/j.agsy.2019.102713

[CR12] Krupová Z, Krupa E, Zavadilová L, Kašná E, Žáková E (2020) Current challenges for trait economic values in animal breeding. Czech J Animal Sci 65:454–462. 10.17221/161/2020-CJAS

[CR13] López-Paredes J, Jiménez-Montero JA, Pérez-Cabal MA, González-Recio O, y Alenda R (2017) A bio-economic model to improve profitability in a large national beef cattle population. Spanish J Agri Res 15(3):e0406. 10.5424/sjar/2017153-10901. (11 pages. eISSN: 2171–9292)

[CR14] Lush JL (1937) Animal Breeding Plans. Iowa State Press, Ames, Iowa

[CR15] Marote ML (2010) Agricultura de precisión, Ciencia y Tecnología 10 ISEU 2010, Repositorio de la Universidad de Palermo

[CR16] McClearn B, Shalloo L, Gilliland TJ, Coughlan F, McCarthy B (2020) An economic comparison of pasture-based production systems differing in sward type and cow genotype. J Dairy Sci 103:4455–4465. 10.3168/jds.2019-17552 32147257 10.3168/jds.2019-17552

[CR17] Mestra-Vargas LI, Barragán-Hernández WA, Medina-Herrera DA, Flórez-Díaz H (2020) Evaluación técnica-económica de la frecuencia de suplementación de novillos en pastoreo en Córdoba, Colombia1. Agronomía Mesoamericana 31(2):353–366. 10.15517/am.v31i2.38389

[CR18] Misztal I, Tsuruta S, Lourenco D, Aguilar I, Legarra A y Vitezica Z. 2018. Manual for BLUPF90 family of programs. University of Georgia, Giorgia, USA. http://nce.ads.uga.edu/wiki/lib/exe/fetch.php?media=blupf90_all7.pdf

[CR19] Mosnier CJ, Agabriel M, Lherm A, Reynaud. (2009) A dynamic bio-economic model to simulate optimal adjustments of suckler cow farm management to production and market shocks in France, Agricultural Systems, 102(1–3). ISSN 77–88:0308-521X. 10.1016/j.agsy.2009.07.003

[CR20] National Research Council -NRC (2021) Nutrient Requirements of Dairy Cattle, Eighth Revised Edition. The National Academies Press, Washington. 10.17226/25806 38386771

[CR21] Notter DR (2013) Importance of genetic improvement programs and breed resources for livestock production systems, XL Reunión de la Asociación Mexicana para la Producción Animal y Seguridad Alimentaria, A.C. (AMPA) y IX Seminario Internacional de Ovinos en el Trópico. researchgate.net

[CR22] Parra-Cortés R, Magaña-Magaña M (2021) Características técnico-económicas de sistemas de producción bovina de las razas criollas colombianas Romosinuano y Hartón del Valle. Revista MVZ Córdoba 26(2):e2079. 10.21897/rmvz.2079

[CR23] Plan Estratégico de la Ganadería Colombiana-PEGA 2019 © (2006) Federación Colombiana de Ganaderos-FEDEGAN. repository.agrosavia.co

[CR24] Pravia MI, Ravagnolo O, Urioste JI, Garrick DJ (2014) Identification of breeding objectives using a bioeconomic model for a beef cattle production system in Uruguay. Livest Sci 160:21–28

[CR25] Ramsey R, Doye D, Ward C, McGrann J, Falconer L, Bevers S (2005) Factors Affecting Beef Cow-Herd Costs, Production, and Profits. J Agric Appl Econ 37:91–99. 10.1017/S1074070800007124

[CR26] R Core Team (2023). R: A language and environment for statistical computing. R. Foundation for Statistical Computing, Vienna, Austria. URL https://www.R-roject.org/

[CR27] Roberts AJ, Petersen MK, Funston RN (2015) Beef Species Symposium: ¿Can we build the cowherd by increasing longevity of females? J Anim Sci 2015(93):4235–4243. 10.2527/jas2014-8811 10.2527/jas.2014-8811 26440322

[CR28] Rowan T (2022) Facilitating profit-driven breeding for commercial cow-calf producers. University of Tennessee Institute of Agriculture. AABP Recent graduate conference Proceedings 55(1):56–57

[CR29] Spangler M (2015) Economically relevant traits and selection indices. Range Beef Cow Symposium. Paper 339. http://digitalcommons.unl.edu/rangebeefcowsymp/339

[CR30] Spangler ML, Weaber RL (2017) Genetic selection vs. Visual appraisal: is it a conundrum? XXV Ranger beef cow Symposium. Cheyenne, Wyoming, November 28. https://beef.unl.edu/

[CR31] Twomey AJ, Cromie AR (2023) Impact of age at first calving on performance traits in Irish beef herds. J Anim Sci 101:1–7. 10.1093/jas/skad008 10.1093/jas/skad008 PMC9907750 36617256

[CR32] Valera Villegas MA, Morillo Moreno MC (2009) Un sistema de costos basado en actividades para las unidades de explotación pecuaria de doble propósito. Caso: Agropecuaria El Lago, S.A. Innovar 19(35):99–177

[CR33] Wahinya PK, Jeyaruban MG, Swan AA, van der Werf JHJ (2022) Breeding objectives for dairy cattle under low, medium and high production systems in the tropics. Animal 16(5):100513. 10.1016/j.animal.2022.100513 35436647 10.1016/j.animal.2022.100513

[CR34] Zuliani A, Mair M, Kraševec M, Lora I, Brscic M, Cozzi G, Leeb C, Zupan M, Winckler C, Bovolenta S (2018) A survey of selected animal-based measures of dairy cattle welfare in the Eastern Alps: Toward context-based thresholds. J Dairy Sci 101(2):1428–1436 29224861 10.3168/jds.2017-13257

